# Mindfulness Training for People With Dementia and Their Caregivers: Rationale, Current Research, and Future Directions

**DOI:** 10.3389/fpsyg.2018.00982

**Published:** 2018-06-13

**Authors:** Lotte Berk, Franca Warmenhoven, Jim van Os, Martin van Boxtel

**Affiliations:** ^1^Department of Psychiatry and Neuropsychology, School for Mental Health and Neuroscience, Maastricht University Medical Centre, Maastricht, Netherlands; ^2^Department of Educational Development and Research, Faculty of Health, Medicine and Life Sciences, Maastricht University, Maastricht, Netherlands; ^3^King’s Health Partners, Department of Psychosis Studies, Institute of Psychiatry, King’s College London, London, United Kingdom

**Keywords:** mindfulness, MBSR, older adults, dementia, caregivers

## Abstract

The world population is aging and the prevalence of dementia is increasing. By 2050, those aged 60 years and older are expected to make up a quarter of the population. With that, the number of people with dementia is increasing. Unfortunately, there is no cure for dementia. The progression of symptoms with no hope of improvement is difficult to cope with, both for patients and their caregivers. New and evidence-based strategies are needed to support the well-being of both caregiver and patient. Mindfulness training is a body-mind intervention that has shown to improve psychological well-being in a variety of mental health conditions. Mindfulness, a non-judgmental attention to one’s experience in the present moment, is a skill that can be developed with a standard 8-week training. Research has shown preliminary but promising results for mindfulness-based interventions to benefit people with dementia and caregivers. The aim of this review is (a) to provide a rationale for the application of mindfulness in the context of dementia care by giving an overview of studies on mindfulness for people with dementia and/or their caregivers and (b) to provide suggestions for future projects on mindfulness in the context of dementia and to give recommendations for future research.

## Introduction

The world population is aging and the prevalence of dementia is increasing. By 2050, those aged 60 years and older are expected to make up almost a quarter of the world’s population (Prince et al., 2013). People who develop dementia mainly express concerns about memory loss, but also experience difficulties with communication, loss of control, and autonomy. Informal caregivers have an important role in the care for people with dementia (de Vugt and Verhey, 2013). Informal caregivers of people with dementia experience providing care as stressful, and show higher levels of psychological distress than caregivers of physically frail elderly people and non-caregivers (Pinquart and Sörensen, 2003). From the moment of the diagnosis of dementia, both the person with dementia and the caregiver will enter a time with stress and uncertainties.

Current interventions to support people with dementia and caregivers often focus on one or the other group, rather than the dyad (Schulz et al., 2007). New strategies are needed to support the well-being of both caregiver and person with dementia. Mindfulness training is an intervention that has shown to improve psychological well-being in both healthy and clinical populations (Fjorback et al., 2011; Hempel et al., 2014; Khoury et al., 2015). Mindfulness based interventions (MBIs) have received increasing empirical support, suggesting it increases well-being of older adults and caregivers in particular.

The aim of this review is (a) to provide a rationale for the application of mindfulness training in the context of dementia care by giving an overview of studies on mindfulness for people with dementia and/or their caregivers, and (b) to provide suggestions for future projects on mindfulness in the context of dementia and to give recommendations for future research.

### Mindfulness-Based Interventions: General Overview

Although there is a wide variety of interventions that include components of mindfulness (e.g., Acceptance and Commitment Therapy), this review focuses on the two programs with the largest evidence base, the mindfulness-based stress reduction (MBSR) and mindfulness-based cognitive therapy (MBCT). These group-based programs have been studied in healthy populations and in those with mental or physical disorders, showing satisfactory to good efficacy (Chiesa and Serretti, 2009; Hofmann et al., 2010; Hempel et al., 2014).

The main characteristic of these standardized programs is the cultivation of mindfulness: being able to direct attention in the present moment, in a non-judgmental way, to allow one to act with awareness. Mindfulness is both a characteristic with a population distribution and a skill that can be trained by practicing mindfulness meditation (Brown and Ryan, 2003). The standard program consists of eight weekly group meetings with a duration of 2–2.5 h each plus a 1-day class during the sixth week, and homework for approximately 45 min a day. The programs incorporate meditative exercises such as the body scan, hatha yoga, sitting meditation, and walking meditation (Kabat-Zinn, 1990; Segal et al., 2002). As with any skill, it is important that participants continue the practice after the program has ended, so that they can continue to develop mindfulness and might use it in future difficult situations.

### Mindfulness and Aging

Recent research on MBI and aging show positive effects on cognitive, emotional, and psychological domains. There is preliminary evidence that mindful meditation may improve attention, memory, executive function, processing speed, and general cognition (Gard et al., 2014; Marciniak et al., 2014). Moreover, studies show that meditation influences brain structure and function, particularly in areas involved in attentional control, self-awareness, and emotion-regulation (Luders, 2014; Boccia et al., 2015; Tang et al., 2015). Although more systematic research is needed, preliminary results indicate that meditation might reduce age-related cognitive decline (Kurth et al., 2017). These conclusions are drawn from both cross-sectional studies (e.g., comparing meditators versus controls) and longitudinal studies with differences in duration and type of meditation training.

Several mechanisms have been proposed by which mindfulness may promote healthy aging. These include enhanced attentional control, preserved neural functioning, improved psychological well-being, and reduced systemic inflammation (Larouche et al., 2014; Fountain-Zaragoza and Prakash, 2017).

The Mindfulness-to-Meaning Theory proposes a mechanism through which mindfulness increases psychological well-being (Garland et al., 2015b). Through mindfulness practice, participants develop a non-judgmental state of present-moment awareness, which has an effect on the interpretations of stressful life events. This developed broadened awareness, mental flexibility, and the ability to see distressing thoughts and emotions as events that will pass, instead of being the truth, reduces the negative impact of stressful events. Promoting positive reappraisal activates an upward spiral of positive psychological processes as specified in the broaden-and-build theory (Fredrickson, 1998), which increases psychological well-being (Garland et al., 2015a). Support for an upward spiral where state mindfulness and reappraisal enhance each other has been found in several studies such as a study using autoregressive latent trajectory modeling (Garland et al., 2017). Another study showed an upward spiral in which state mindfulness and positive affect enhanced each other (Gotink et al., 2016).

Moreover, the reduction of stress may result in increased telomerase activity, lower blood pressure, and heart rate (Innes and Selfe, 2014). Furthermore, a recent review suggests that mindfulness meditation may increase cognitive reserve capacity and may mitigate age-related cognitive decline (Malinowski and Shalamanova, 2017).

These mechanisms have been proposed as a general pathway to possibly reduce the risk of developing dementia and to slow the process of neurodegeneration. Although studies on improving cognition in older adults with MBI are inconclusive (Berk et al., 2016), there is preliminary evidence that MBI increases psychological well-being in older adults, although not without limitations (Geiger et al., 2016). The majority of this support comes from feasibility studies without a comparison group. These studies reported benefits on pain, attention, sleep, mood elevation, and global quality of life (Morone et al., 2008), emotional well-being (Splevins et al., 2009), anxiety, ruminative thoughts, sleep problems, depressive symptoms (Foulk et al., 2014), and emotional distress (Young and Baime, 2010). One of the two randomized controlled trials (RCT) in the area of aging reported greater reductions in loneliness in the MBSR participants than the wait-list group (Creswell et al., 2012). The other RCT reported improvement on disability, pain severity, and psychological functioning for both the MBSR and education group (Morone et al., 2009).

In the following sections, we provide a review of research on MBI for caregivers (section “Mindfulness and Caregivers”) and patient and caregiver dyads (see section “Mindfulness for Caregiver and Patient Dyads”). In the sections thereafter we focus on research involving people with dementia with a review of research on MBI for people with dementia (see section “Mindfulness for Persons with Dementia”), caregivers of people with dementia (see section “Mindfulness for Caregivers of Persons with Dementia”), and people with dementia and caregiver dyads [see section “Mindfulness for Dyads (Dementia and Caregivers Together”)]. Studies were identified by searches of PubMed, Web of Science, and PsychINFO using the following search terms: “(mindfulness OR MBCT OR MBSR)” AND “(dementia OR Alzheimer OR cognitive decline OR MCI OR caregivers).” Studies on mild cognitive impairment (MCI) and subjective cognitive decline (SCD) were included since individuals with these conditions have a high risk to develop dementia. Both qualitative and quantitative studies were included, and studies needed to include a standard MBI. In addition, additional relevant studies were identified from the reference lists of examined articles.

### Mindfulness and Caregivers

Research on MBI to support informal caregivers suggests feasibility and potential benefit on mental health (Jaffray et al., 2016; Li et al., 2016). Most research involved uncontrolled trials for caregivers of a variety of conditions, ranging from terminally ill patients (e.g., patients with advanced-stage cancer) to chronic conditions (e.g., diabetes).

An uncontrolled pilot study with caregivers of elderly patients with cognitive or other functional impairment underwent an adjusted MBSR program (with shorter 90-min sessions) and showed improvements in depression symptoms, but no improvements in mindfulness (Epstein-Lubow et al., 2011). One RCT on caregivers of patients with chronic conditions showed an improvement on depression, which was the primary outcome, compared to an active control (Hou et al., 2014). Moreover, an improvement in anxiety, self-efficacy, and mindfulness were found. However, no effects were found on stress, quality of life, or self-compassion.

### Mindfulness for Caregiver and Patient Dyads

Because the lives of caregivers and patients are closely related and MBI aims at being present without judgment with these (shared) life experiences, it may be that the beneficial effects of training could be enhanced by applying the intervention to the system of both patient and caregiver. There are several uncontrolled studies that recruited caregivers and patients to participate in the MBI together. In one pre-test and post-test design study, 21 patient-caregiver dyads completed an MBSR training and both caregiver and patients showed improvement in mood, stress, and mindfulness (Birnie et al., 2010a). A pilot study with an adapted MBSR (6 weeks: three in-person sessions, three audiotaped sessions) for caregivers of cancer patients included 26 dyads and reported improvement of stress and anxiety for patients, but no significant changes for the caregivers (Lengacher et al., 2012). A mixed-method study included 19 lung cancer patients and 16 partners (van den Hurk et al., 2015). Caregiver burden decreased among the partners. However, no significant changes were found in mental distress among all participants. The qualitative analysis of the semi-structured interviews showed that the MBSR training started a process in which both patients and partners became more aware of, and gained more insight into, their thoughts, feelings, and bodily sensations. Moreover, participants reported that it was helpful to participate together with their partner. They encouraged each other to perform the exercises, and it led to better mutual understanding.

## Mindfulness and Dementia

### Mindfulness for Persons With Dementia

Studies with persons with MCI or SCD have looked at the effect of MBI. This is informative for dementia research, since individuals with MCI have an increased annual conversion rate of 5–17% to Alzheimer’s disease (Cheng et al., 2017), and approximately 60% over a 15-year period of persons with SCD will continue to develop Alzheimer’s disease (Reisberg et al., 2008).

Studies with persons with MCI or subjective memory complaints have looked at the effect of MBI. One pilot study was a randomized trial with 14 people with MCI, which found a trend toward improvement of cognition, quality of life, and well-being for people in the mindfulness condition (Wells et al., 2013). An RCT with 22 people of MCI, showed that the participants in the MBI group showed less memory deterioration and greater decrease in depressive symptoms compared to the control group (Larouche et al., 2016).

Besides these studies on MCI, three other studies involved persons with SCD. One pilot study included 34 older adults with SCD and showed improved worry severity (Lenze et al., 2014). Another study on MBSR for people with SCD involved an RCT that included 14 older adults with and 22 without SCD (Smart et al., 2016). The participants with SCD reported a decrease in cognitive complaints and increase in memory self-efficacy. Attention regulation was improved in all MBSR participants. Recently, a feasibility mixed-methods study in middle-aged and older adults with SCD reported that participants after MBSR worried less about their memory complaints (Berk et al., 2017). Moreover, an RCT with older adults with stress disorders and subjective neurocognitive problems compared MBSR to health education, with the primary outcomes being memory and cognitive control (Wetherell et al., 2017). Participants receiving MBSR showed greater improvement in memory, but not cognitive control. Moreover, the MBSR group improved on measures of worry, depression, and anxiety, and decreased cortisol level for those with high baseline cortisol.

Although these studies demonstrate feasibility of MBSR with older adults with SCD and MCI, and preliminary evidence for memory improvement, more research is necessary to investigate whether MBI can influence cognitive decline. Such studies are beginning to be developed. Recently, a protocol mixed-methods longitudinal study for mindfulness with persons with MCI was published. This study will investigate whether a customized MBI will improve cognitive function, mental health, mindfulness, and functional abilities in daily life activities of persons with MCI, with a 1-year follow-up (Wong et al., 2016).

### Mindfulness for Caregivers of Persons With Dementia

A recent systematic review reported that caregivers of persons with dementia show elevated stress, and poorer attention and executive function performance (Allen et al., 2017). Moreover, caregivers of persons with dementia show increased levels of anxiety and depression (Baumgarten et al., 1994; Mahoney et al., 2005). Studies have reported that caregiving for a person with dementia may be particularly stressful compared to other forms of caregiving (Ory et al., 1999; Kim and Schulz, 2008).

Three RCTs have investigated the effects of MBI in caregivers of persons with dementia (Oken et al., 2010; Whitebird et al., 2012; Brown et al., 2016). One study divided 31 caregivers between an adapted MBCT (90 min sessions, 7 weeks) and two control groups: education and respite only (Oken et al., 2010). Both MBCT and education improved the primary outcome of caregiver stress compared to the respite-only group. No effects were found on the secondary outcome measures of cognition and mindfulness. Another study randomized 78 caregivers of persons with dementia into a MBSR or active control group (Whitebird et al., 2012). Caregivers in the MBSR group showed greater improvement in overall mental health, stress, and depression. Both interventions improved anxiety, social support, and burden. In another RCT, 38 family caregivers of persons with dementia were randomized to MBSR or standard social support control condition (Brown et al., 2016). The caregivers in the MBSR group reported lower levels of perceived stress relative to active control group, but not at the 3-month follow-up.

### Mindfulness for Dyads (Dementia and Caregivers Together)

To date, only two studies have investigated MBI including both persons with dementia as well as their caregivers. One study with single-group, pre-test and post-test design, involved an adjusted MBSR for persons with progressive cognitive decline (*n* = 17, majority with dementia) and 20 caregivers (Paller et al., 2014). This study showed improvement in quality of life and depressive symptoms, but no significant findings on cognitive functioning for both patients and caregivers. Another pilot study involved 12 persons with dementia and 8 caregivers with a standard MBSR (Leader et al., 2013). Interviews and observations indicated that some participants with dementia were able to learn mindfulness and experienced increases in quality of life. The caregivers were very positive in their evaluation in that it was helping them to cope better with life. Both groups showed improved quality of life after the intervention, but this was not maintained at the 3-month follow up.

These studies show that it is feasible and potentially beneficial to involve the person with dementia and their caregivers together in an MBI. Although the majority of studies have separated these groups, the well-being of a caregiver and the person with dementia should be considered in context because they influence each other. One study using both cross-sectional and longitudinal analyses investigated the role of suffering in 1222 persons with dementia and the well-being of their caregivers (Schulz et al., 2008). This study showed, in both cross-sectional and longitudinal analyses that perceived suffering of the person with dementia contributes to caregiver depression and burden. Importantly, this was after controlling for the effects of cognitive and physical disability, memory problems, disruptive behaviors, and time spent on caregiving.

## Future Directions and Considerations

Although current research supports the rationale for MBI with persons with dementia and their caregivers, only few RCTs have been conducted and more research is necessary (see **Table 1**). There are several recommendations for future research.

**Table 1:** Overview of MBI studies with caregiver and patient dyads, and MBI studies with persons with SCD, MCI or dementia.

Author (year)	Design	Sample	Care recipient relationship	Adjustments to MBSR/MBCT	Outcome Measures	Results
Berk et al. (2017)	UCT	Older adults with SCD (*n* = 13), age (*M* = 59, range 45–85), 54% male	NA	8 weekly sessions, 2.5-h, day long retreat.	FFMQ-SF, SCS-SF, EuroQol, DASS-21, MIA, VLT, Trail Making Test Aand B	Improved verbal memory (delayed recall). Participants reported positive effects; e.g., worried less about memory complaints.
Birnie et al. (2010a)	UCT	Cancer patients and their partners (*n* = 82) 47.6% male, age patients (*M* = 62.9, *SD* = 7.37), age partners (*M* = 62.8, *SD* = 9.34)	Spouse (*n* = 20) Common-law (*n* = 1)	No details	POMS, C-SOSI, MAAS	Significant reductions in mood disturbance, muscle tension, neurological/GI, and upper respiratory symptoms. Partner’s mood disturbance scores were positively correlated with patients’ symptoms of stress and negatively correlated with patients’ levels of mindfulness
Brown et al. (2016)	RCT	Caregivers of family members with dementia, *n* = 38 (MBSR = 23, CON = 15), age (*M* = 61.14, *SD* = 10.41), 15.8% male, CON = social support	Parent (*n* = 19)Spouse (*n* = 16)Other (*n* = 3)	8 weekly sessions, 1.5–2-h, day long retreat.	PSS, AAQ, POMS, SF-36, ZBI, FCI-MS, salivary cortisol.	MBSR lower levels of perceived stress and mood disturbance post-intervention compared to CON, at 3 month FU no difference between groups. No differences in diurnal cortisol response change of the course of the study
Larouche et al. (2016)	RCT	Older adults with MCI, *n* = 22 (MBI = 11, CON = 11), age (*M* = 71.6, *SD* = 7.6), 63.6% male	NA	NS	Verbal free-recall memory test, geriatric depression scale, WHO quality of life.	Post-intervention, MBI less objective memory deteriorations, greater decrease in depressive symptoms, increased quality of life
Leader et al. (2013)	UCT	People with dementia (*n* = 12) and caregivers (*n* = 8)	NS	8 weekly sessions, 2.5 h.	WEMWBS, qualitative	Increase in WEMWBS, not maintained after 3 month FU. Interviews showed people with dementia able to learn mindfulness and experienced increased quality of life; the caregivers evaluated positively and helped with coping.
Lengacher et al. (2012)	UCT	Cancer patients and their family caregivers (*n* = 52)	Family caregivers: 53.8% lives with patient	Modified for 6-week program. Three in-person sessions and three audiotaped sessions (content validity index score of 0.944), 2-h sessions	PSS, CESDS, STAI, MSAS, MOS-SF-36, Cortisol and immune (IL-6)	Post interventions, patients improved on stress and anxiety. Both patients and caregivers lower IL-6
Lenze et al. (2014)	UCT	Two MBSR groups, *n* = 34. 8-week program (*n* = 16), 12-week program (*n* = 18), 12-week age (*M* = 70.7, *SD* = 4.9) 22% male, 8-week age (*M* = 70.9, *SD* = 4.5) 31% male	NA	8 weekly sessions, 2.5 h. sessions and daylong session. 12-week reduced day-long retreat to 2.5 h.	List learning, digit span test, verbal fluency, color-word interference, PSWQ-A, CAMS-R, MAAS	Sign. improvement post-intervention List, delayed recall, Paragraph immediate recall, Paragraph delayed recall, Verbal Fluency Color-word interference, worry reduction (PSWQ-A), mindfulness (measured by CAMS-R, but not MAAS),
Oken et al. (2010)	RCT	Three groups: MBCT (*n* = 10), age (*M* = 62.50, *SD* = 11.61) 20% male, education class (EDU), *n* = 11, age (*M* = 67.09, *SD* = 8.36), 27% male; respite only (CON), *n* = 10, age (*M* = 63.80, *SD* = 7.93), 10% male	Parent (*n* = 8)Spouse (*n* = 23)	6-weekly 90-min group sessions	RMBPC, PSS, CESD, SF-36 Fatigue, MAAS, FFNJ, GPSE, ESS, NPI total, Caregiver appraisal, CRI, Cortisol, IL-6, TNF-α, hsCRP, Stroop interference, ANT, Word list immediate and delayed recall.	Improved caregiver self-efficacy (RMBPC) for both MBI and EDU compared to CON
Paller et al. (2014)	UCT	People with progressive cognitive decline (=17) and caregivers (*n* = 20), 19 were part of patient-caregiver dyad. Age patients (*M* = 72), age caregivers (*M* = 62.5). Diagnosis patients: Dementia (AD: *n* = 9, FTD: *n* = 1), MCI (*n* = 2), memory loss due to strokes (*n* = 2), memory complaints (*n* = 3)	Parent (*n* = 5)Spouse (*n* = 13)Other (*n* = 2)	8 weekly sessions, 1.5 h. Elements of dialectical behavior therapy and acceptance and commitment therapy	QOL-AD, GDS, PSQI, BAI, Trail Making Test A and B, RBANS,	Increased quality of life, fewer depressive symptoms, better subjective sleep quality.
Smart et al. (2016)	RCT	Older adults with (*n* = 14), mean age 70.0 (*SD* = 3.45, 61% male) and without SCD (*n* = 22), mean age 69.6 (*SD* = 3.58, 27% male) randomized into MBI or psychoeducation	NA	8 weekly sessions, 2 h.	CCI, MSEQ, FFMQ	SCD participants showed decrease in cognitive complains and increase in memory self-efficacy after intervention. Attention regulation was improved in MBSR participants.
van den Hurk et al. (2015)	UCT	Lung cancer patients (*n* = 19, mean age 61.7, range 54–77, 53% male) and partners (*n* = 16, mean age 60.9, range 30–76, 44% male)	Partners (*n* = 16)	8 weekly sessions, 2.5 h. sessions and day long retreat.	HADS, QLQ-LC, CIS-F, IES, PSWQ, MAAS, SPPIC, CRA-SE	Caregiver burden in partners decreased after MBSR.
Wetherell et al. (2017)	RCT	Older adults with SCD randomized to MBSR (*n* = 47, mean age 70.4, *SD* = 4.1, 13% male) or health education control condition (*n* = 56, mean age 73.3, *SD* = 6.1, 27% male)	NA	8 weekly session, 1.5 h., half-day meditation retreat.	Memory (immediate, delayed paragraph and list recall), Verbal Fluency Test and Color Word Interference Test (DKEFS), PSWQ, CGI-I, PROMIS, CAMS-R, cortisol	MBSR group improved on worry, depression symptoms at post intervention and worry, depression and anxiety symptoms. MBSR participants showed decreased cortisol level for those with high baseline cortisol.
Wells et al. (2013)	RCT	Older adults with MCI (*n* = 14) randomized into MBSR, age (*M* = 73, *SD* = 8) or CON, age (*M* = 75, *SD* = 7).	NA	8 weekly sessions, 2 h, and one daylong retreat day.	ADAS-cog, RAVLT, COWAT, RS, PSS, QOL-AD, HHI, LOT-R, CES-D, MAAS	Control subjects performed better than the MBSR group on the Trails A and B tests. Non-significant trends that suggested improvement with MBSR vs. control were detected for quality of life, cognition, and well-being.
Whitebird et al. (2012)	RCT	Caregivers of family members with dementia randomized in MBSR [*n* = 38, age (*M* = 57.2, *SD* = 9.6), 13.2% male] or active control [CCES: *n* = 40, age (*M* = 56.4, *SD* = 10.2), 10% male]	Parent (*n* = 58)Spouse, sibling or friend (*n* = 20)	8-week, 2.5-h, and a 5-h retreat day.	PSS, CESD, STAI, MBCBS, MOSSSS	MBSR improved overall mental health, reduced stress, decreased depression compared to CCES. Both interventions improved caregiver mental health and improved anxiety, social support and burden.

*AAQ, Acceptance and Action Questionnaire II; ADAS-cog, Alzheimer’s Disease Assessment Scale cognitive subscale; ANT, Attentional Network Test; BAI, Beck Anxiety Inventory; CAMS-R, Cognitive Affective Mindfulness Scale-Revised; CESD, Center for Epidemiologic Studies Depression Scale; CIS-F, Checklist Individual Strength-Fatigue; CON, control group; COWAT, Controlled Oral Word Association Test; CRA-SE, Caregiver Reaction Assessment-Care-derived Self-Esteem; CRI, Coping Responses Inventory; C-SOSI, Calgary Symptoms of Stress Inventory; ESS, Epworth Sleepiness Scale; FCI-MS, Mutuality Scale of the Family Care Inventory; FFNJ, Five Factor Mindfulness Questionnaire Non-judgment Subscale; FU, follow-up; GDS, Geriatric Depression Scale; GPSE, General Perceived Self-Efficacy; HADS, Hospital Anxiety and Depression Scale; HHI, Herth Hope Index; hsCRP, high-sensitivity C-reactive protein; IES, Impact of Event Scale; IL-6, interleukin-6; LOT-R, Life Orientation Test-Revised; MAAS, Mindful Attention Awareness Scale; MBCBS, Montgomery Borgatta Caregiver Burden Scale; MBCT, mindfulness-based cognitive therapy; MBSR, mindfulness-based stress reduction; MBI, mindfulness based intervention; MCI, mild cognitive impairment; MOSSSS, Medical Outcomes Study Social Support Survey; NA, not applicable; NPI, Neuropsychiatric Inventory; NS, not stated; POMS, Profile of Mood States; PSQI, Pittsburg Sleep Quality Inventory; PSS, Perceived Stress Scale; PSWQ-A, Penn State Worry Questionnaire-Abbreviated; PSQI, Pittsburgh Sleep Quality Index; QLQ-LC, Quality of Life Questionnaire – Lunch Cancer; QOL-AD, Quality of Life-Alzheimer’s Disease; RAVLT, Rey Auditory Verbal Learning Test; RBANS, Repeatable Battery for the Assessment of Neuropsychological Status; RMBPC, Revised Memory and Behavior Problems Checklist; RS, resilience scale; SCD, subjective cognitive decline; SD, standard deviation; SPPIC, Self-Perceived Pressure from Informal Care; TNF-α, tumor necrosis-α; WEMWBS, Warwick Edinburgh Mental Well-being Scale; UCT, uncontrolled trial; ZBI, Zarit Burden Interview*.

First, more methodologically rigorous trials (RCT with active control) are recommended. The active control is a particularly important component given the strong evidence of the Dodo bird verdict; that all psychotherapies are equally effective (Luborsky et al., 2006; Cuijpers and Pim, 2017). However, this does not mean that the effects are realized by the same mechanisms. Since a mindfulness intervention is a complex process, research projects should not only consider the outcomes of a trial, but also address the mechanisms by which the effects are realized to gain more insight into applying mindfulness in the complex setting of a person with dementia and caregiver. Also, not much is known about the long-term effects, therefore follow-up measurements are recommended. Moreover, an active control for MBI should particularly control for the non-specific effect of “community” associated with mindfulness training. That is, the active control condition needs to provide the same degree of personal contact, being together with other people and feeling of “community.”

Second, it is unclear if and what kinds of adjustments are necessary for people with dementia and their caregivers. In several studies, adaptation of the MBSR or MBCT protocol to the context of a person with dementia and caregiver is frequently practiced, but it is unclear what the effects of specific adaptations are or what the rationale is. For example, many studies adjust the standard MBSR or MBCT protocol to reduce the duration of the sessions (e.g., Ernst et al., 2008), the duration of homework (e.g., Mallya and Fiocco, 2015), or do not include a silent day (e.g., Paller et al., 2014). Another study increased the duration by spreading out the 8 sessions over 8 months (Zellner Keller et al., 2014). However, one study compared an extended 12-week MBSR to an 8-week MBSR in older adults with worry symptoms and subjective cognitive dysfunction, and they found no difference in effects for the different duration of the MBSR (Lenze et al., 2014). Another study showed that a shorter 4-week MBSR with 1-h sessions improved caregiver burden (Hoppes et al., 2012). With different adjustments and different outcome measures it is difficult to tell which elements are crucial. Moreover, other programs that have major adaptations to an MBSR or MBCT protocol have been developed for people with dementia (Chan et al., 2017), and a pilot study showed its feasibility in care homes (Churcher Clarke et al., 2017). Other longer forms showed benefits on cognition in people with Alzheimer’s disease over a 2-year period, including three weekly sessions based on mindfulness, cognitive stimulation therapy, and progressive muscle relaxation (Quintana-Hernández et al., 2015).

Instead of general adjustments, there may be large inter-individual differences driving the need for adjustment within the training group. Therefore, it might be useful to approach the training with a certain amount of flexibility, both from the trainers and the participants. There might not be a “one size fits all” approach. Keeping that in mind, future research should investigate which modifications are beneficial. For example, practical strategies to support home practice and choice in personalized materials should be considered. Perhaps more exercises dedicated to compassion and self-compassion would be a valuable addition. This, however, is ideally investigated with a three-armed RCT (MBI with and without extra compassion, and an active control). Investigating components of the MBI have recently been a topic of discussion, not just for people with dementia and their caregivers. Recently, essential characteristics of MBI and teachers of MBI have been described (Crane et al., 2017). However, it does not touch upon the optimal amount of practice time in the sessions and time spent on homework assignments. Investigating home-practice could lead to different guidelines (i.e., less time) for homework, since the amount of time practiced seems to not be related to the positive outcomes of the program (Dobkin and Zhao, 2011). These and other studies measuring practice time, rely on self-report. However, similar results were found using objective electronic measurement in a 6-week MBCT-based program that showed that adherence to practice (length, frequency, and type of meditation chosen) did not relate to outcomes (Ribeiro et al., 2017). Recently, a first study to directly compare an 8-week with a 4-week MBI showed improvements compared to controls in mindfulness and positive affect, but not between the 4- and 8-week MBI (Demarzo et al., 2017). Thus, abbreviated MBI might be similar to a standard MBI. These studies on length of the program and practice might guide future research on adjustment of the program. A shorter program or shorter sessions might be just as effective, and would make it more accessible for a larger number of people. In particular, time investment could be a major obstacle for caregivers with people with dementia, and a shorter program could increase the likelihood of participation.

Third, future research could investigate whether it is desirable to have people with dementia and their caregiver participate in the MBI together. Although it seems feasible (e.g., Paller et al., 2014), caregivers may feel conflicted when they are invited to pay attention to themselves and focus on their own needs, preventing them from fully benefitting. For example, a few of participants in a study with MBSR for couples facing lung cancer indicated that they felt worried and distracted about the well-being of their partner (van den Hurk et al., 2015). Moreover, caregivers might be reluctant to discuss their fears. Perhaps a program that allows the dyad to separate at specific times could be investigated.

Fourth, future research should consider the following outcome measures to gain more insight into the effects of the MBI. Perhaps it is more fruitful to not only focus on cognition but also on other aspects, such as positive health (Huber et al., 2016). Measuring the influence of compassion on the relationship of the caregiver and person with dementia could be investigated since MBI can improve compassion and that, in turn, might improve couple functioning. One dyadic study with lung cancer patients and their partners showed that more self-compassion was related to less psychological distress in individuals with low levels of self-compassion (Schellekens et al., 2017). Unfortunately, compassion was not measured in most studies. However, research has shown self-reported increases in self-compassion after meditation, impacting mental health (Birnie et al., 2010b). Increasing self-compassion among persons with dementia and caregivers might be particularly valuable since it helps to buffer against anxiety and is associated with increased well-being (Neff et al., 2007). It thus may boost personal resources to supply care for others. In addition, meditation enhances compassionate responding; thus, acting to relieve a person’s suffering (Condon et al., 2013; Lim et al., 2015). Increased caregiver compassion could support the person with dementia. Measuring caregiver management strategies and the impact of MBI might also be interesting for future research since caregivers characterized by non-acceptance was associated with behavioral problems in people with dementia (De Vugt et al., 2004). MBI is thought to increase acceptance and therefore, it might also have an influence on the caregiver style. Future studies could investigate cost-effectiveness in order to make the training program more interesting for reimbursement (e.g., via health insurance). Another important direction of future research is to identify, understand, and develop strategies to overcome barriers to participation, such as time, finances and personal convictions; not only at the individual level, but also what the strategies and barriers are to deliver the program on a larger scale.

This review focuses on older adults since dementia is an age-related disease, however, the essential adjustments to the training are not particularly age-driven. That is, MBI would also be suitable for persons with early onset dementia. A major concern in this field of research is that there are many forms of meditation. Although this review is focused on MBSR/MBCT, other programs with a mindfulness component are developed and researched. It is important to investigate the potential mechanisms and carefully consider the reasons for adjustments.

## Conclusion

In sum, current research supports the rationale for MBI with persons with dementia and their caregivers, shows that it is feasible with dyads, and shows preliminary support for improvement in their well-being. In applying interventions for people with dementia and caregivers, their well-being should be considered in context and how dyads influence each other. MBI may offer the couple a skill that they can both use in the future challenges that accompany the context of living with dementia, thus enhancing resilience and autonomy. However, more research is necessary, in particular with respect to what adjustments are beneficial and necessary, and the use of outcomes measures related to positive health.

## Author Contributions

All authors listed have made a substantial, direct and intellectual contribution to the work, and approved it for publication.

## Conflict of Interest Statement

The authors declare that the research was conducted in the absence of any commercial or financial relationships that could be construed as a potential conflict of interest.
